# Targeting transferrin receptor delivery of temozolomide for a potential glioma stem cell-mediated therapy

**DOI:** 10.18632/oncotarget.20165

**Published:** 2017-08-10

**Authors:** Ting Sun, Haibin Wu, Yanyan Li, Yulun Huang, Lin Yao, Xionghui Chen, Xiaoxiao Han, Youxin Zhou, Ziwei Du

**Affiliations:** ^1^ Neurosurgery & Brain and Nerve Research Laboratory, The First Affiliated Hospital of Soochow University, Suzhou, Jiangsu, China; ^2^ Emergency Surgery, The First Affiliated Hospital of Soochow University, Suzhou, Jiangsu, China

**Keywords:** targeting delivery, transferrin receptor, glioma stem cells

## Abstract

Glioma stem cells, which are sub-populations of tumor cells, are responsible for resistant responses to radiotherapy and chemotherapy after surgery. Targeting resistant glioma stem cell sub-populations might present a novel means to prevent tumor recurrence. Due to the high expression of transferrin receptors at the surface of brain capillary endothelial and tumor cells, especially glioma stem cells, targeting the transferrin receptor system provides an avenue for the entry of drug molecules into the brain. Nanoparticles that target glioma stem cell sub-populations, conjugate transferrin and encapsulate temozolomide, were developed as a potential therapeutic strategy to evaluate their effectiveness at damaging tumor cells. Nanoparticles were highly effective at penetrating the blood-brain barrier and delivering a high therapeutic dose of temozolomide. This effective means of delivery provoked enhanced cytotoxicity against glioma cells, and especially against glioma stem cells. The targeting transferrin receptor nanoparticles display an inherent capacity for a highly therapeutic approach in targeting glioma stem cells and non-stem cells tumors. In addition, transferrin nanoparticles encapsulating temozolomide have the potential of a promising anti-tumor strategy against glioma of the O6-methylguanine-DNA-methyltransferase gene promoter methylation.

## INTRODUCTION

Glioma stem cells (GSCs) are a sub-population of stem cells that remain non-proliferative for extended periods that have the capacity to re-enter the cell cycle to reestablish a viable tumor under select microenvironmental conditions [1]. This sub-population of tumor cells is responsible for resistance to radio- and chemotherapy following surgery. Promising data has revealed that targeting resistant GSCs may present a novel approach at blocking tumor recurrence [2, 3].

The grim prognosis of glioblastoma multiforme (GBM) is due in part to structural barriers including the blood-brain barrier (BBB), which prohibits entry of chemotherapeutic agents. Experimental methods aimed at achieving highly effective chemotherapeutic penetration to the site of the tumor, have been a major focus recently, and shown promise in the treatment of malignant diseases of the brain [4]. The BBB comprises brain capillary endothelial cells (BCECs) that predominantly restrict paracellular substrate flux and free exchange of molecules larger than 400 Da. In addition, due to the low permeability of the BBB and expression of transferrin (Tf) receptors (TfR) on the surface of BCECs, targeting the TfR system provides a route that allows the entry of drugs and nanoparticles to the brain [5-6].

Multiple experimental studies have assayed the expression of TfR1 and TfR2, and found that both were increased on both proliferating and malignant cells, including GBM, as compared to normal brain tissue. Clinical studies have revealed that TfR1 expression was correlated with poorer outcomes [7-8]. The expression of TfR was also increased in cancer stem cells (CSCs) as compared non-CSCs. The surface expression levels of TfR were noticeably elevated in CSCs, likely because of increased recycling or enhanced expression of TfR. In addition, TfR was essential in the maintenance of CSC and increased the frequency of CSCs by nearly 10-fold [9]. Thus, targeting of transferrin receptor therapeutics for GSCs has shown promising potential as a novel therapeutic to target GBM.

GBM with the O6-methylguanine-DNA-methyltransferase (MGMT) gene promoter methylation status is sensitive to temozolomide (TMZ) chemotherapy; however, recurrence is inevitable, and is perhaps due to the existence of GSCs, which when present display only partial uptake of TMZ. Clinical samples and GSC cell-lines expressing the methylated MGMT gene promoter were used to evaluate the effects of Tf-targeted nanoparticles pre-loaded with TMZ with the aim of damaging GSCs and inhibiting regrowth of GBM orthotopic xenografts.

## RESULTS

### Identification of PAMAM-PEG–Tf bioconjugates

**Figure 1:**
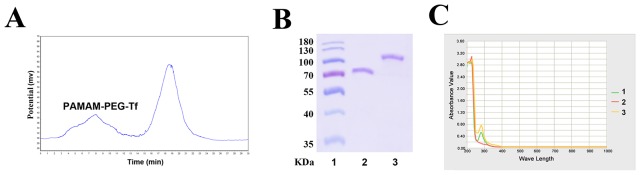
Confirmation and purification of bioconjugate **(A)** Spectra of gel filtration showed the separation of Tf and PAMAM-PEG-Tf. The first peak indicated PAMAM-PEG-Tf, and the second peak indicated unconjugated Tf. **(B)** The conjugation of PAMAM and Tf and the purification of bioconjugated product were observed by SDS-PAGE. Lane 1, Protein ladder; Lane 2, Tf; and Lane 3, PAMAM-PEG-Tf. **(C)** Ultraviolet-visible spectra of PAMAM-PEG-Tf was seen in all band scanning. Characteristic absorbance peak at approximately 280nm indicated the presence of Tf. Lanes 1, Tf; Lane 2, PAMAM; and Lane 3, PAMAM-PEG-Tf.

Bioconjugated products were purified by gel filtration to separate Tf and PAMAM-PEG-Tf, which were indicated by two peaks in the graphical spectra (Figure 1A). PAMAM-PEG-Tf was obtained by SDS-PAGE, which showed a new band at about 110 kDa, which implied a Tf ligand that was covalently attached to PAMAM-PEG. No bands were seen at approximately 80 KDa in the PAMAM-PEG-Tf lane, which strongly supported the success of the purification procedure (Figure 1B). Conjugation of Tf and PAMAM was aided by ultraviolet-visible spectra of all bands scanned. An apparent peak at 280nm from Tf was observed when detecting the biconjugated PAMAM-PEG–Tf, which indicated conjugation of PAMAM and Tf by PEG since no peak was seen in PAMAM (Figure 1C). The results of SDS-PAGE and ultraviolet-visible spectra also showed that one Tf molecule was conjugated to one PAMAM-PEG molecule. The concentration of the nanoparticles PAMAM-PEG–Tf/TMZ was calculated as 200 μM using a Tf standard curve.

### Encapsulating efficiency (EE%) and drug loading capacity (LC%)

According to calculation by free TMZ in solution, the results of TMZ EE% was (74.5 ± 7.8%) in the PAMAM-PEG–Tf and (79.4 ± 2.9 %) in PAMAM. The LC% of TMZ was (14.71 ± 1.29 %) in PAMAM and (4.31 ± 0.35 %) in PAMAM-PEG–Tf. The solubility of TMZ in water was enhanced when it interacted with PAMAM or PAMAM-PEG-Tf. The TMZ concentration of 5.6 mM in PAMAM and 5.2 mM in PAMAM-PEG–Tf solution was calculated. There were no significant differences in terms of percent EE and TMZ concentration between PAMAM and PAMAM-PEG–Tf. However, the percent LC of TMZ in PAMAM-PEG–Tf was significantly lower than that found for PAMAM (*P* < 0.01) due to the presence of Tf.

### Methylation status of the MGMT promoter of GBM

**Figure 2:**
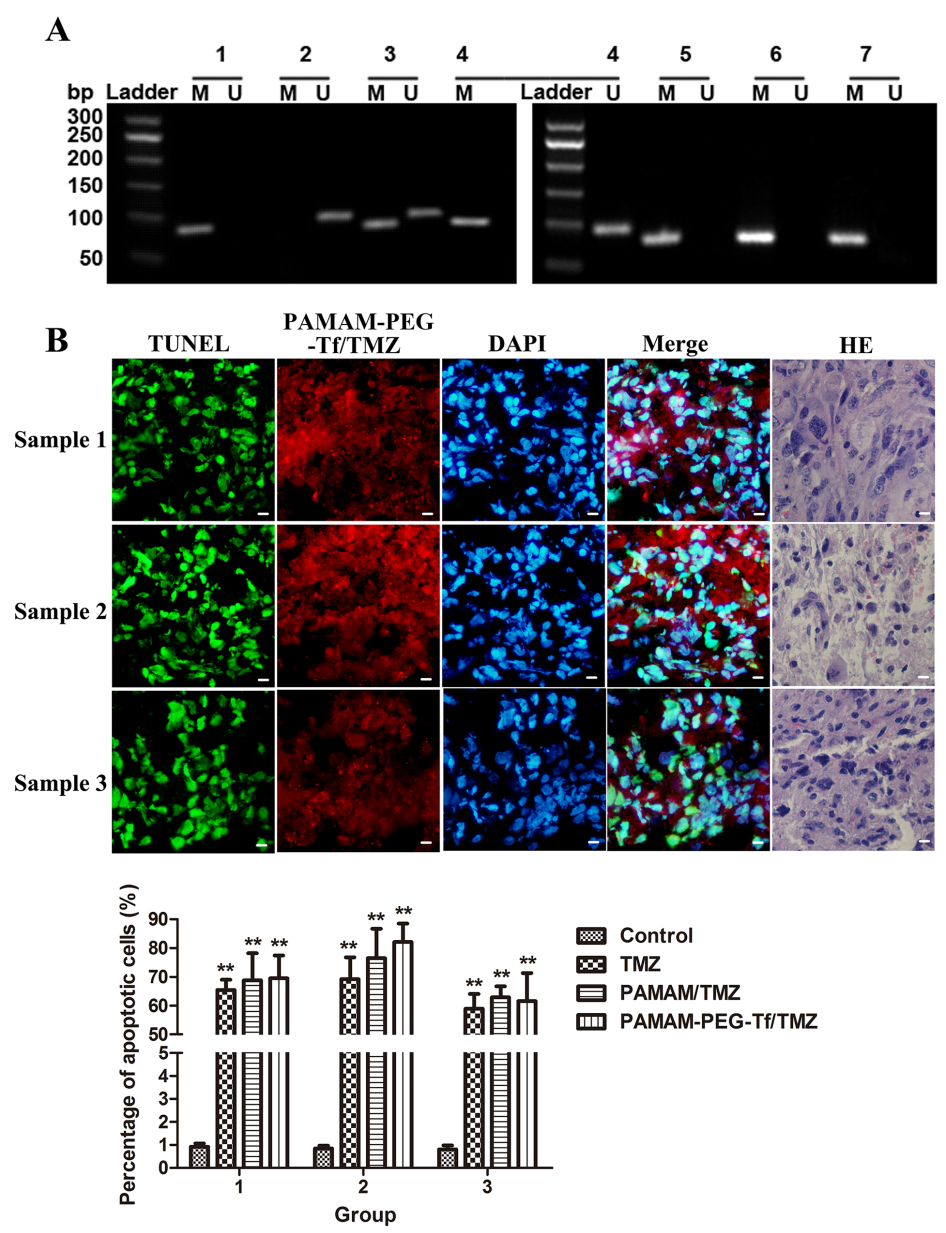
Biological characteristic of sorted GSCs from surgical GBM samples **(A)** Methylation status of the MGMT promoter in GBM resected tissues and cultured GSCs.Fragments of 81bp were amplified in methylated MGMT promoters (M) and 93bp were amplified in unmethylated MGMT promoters (U) using a nested MSP, and were present in all tumor specimens. 1. Methylated DNA control; 2. Unmethylated DNA control; 3. Sample 1; 4. Sample 2; 5. Sample 3; 6. SU2 cells; and 7. 51A cells. A 50bp ladder was loaded to estimate the molecular size, as shown on the left scale. **(B)** The uptake and apoptotic effect of PAMAM-PEG-Tf/TMZ under fluorescent microscopy in surgical samples of glioblastoma showed Grade IV status by histopathology. Red fluorescence was derived from PAMAM-PEG-Tf/TMZ, green fluorescence indicated apoptotic cells, and blue fluorescence showed counter-staining of the nucleus by DAPI (×400 magnification). Scale bar =10 μm. **(C)** The percentage of apoptotic cells in surgical GBM samples after treatment with saline, TMZ, PAMAM/TMZ and PAMAM-PEG-Tf/TMZ. **P < 0.01 vs. control.

Methylation status of the MGMT promoter using a nested Methylation-Specific Polymerase Chain Reaction (MSP) assay in GBM resected tissues are shown in Figure 2A. Both the methylated and unmethylated status of the MGMT promoter were shown for samples 1 and 2, and the positive methylation status was shown in sample 3, as well as for cultured GSCs SU2 and 51A, which were all considered TMZ sensitive in the clinic.

### Cytotoxicity of PAMAM-PEG–Tf/TMZ in GBM surgical samples

Cryostat sections using TUNEL assay was used to determine the apoptotic effect of drugs and then analyzed by fluorescence microscopy after GBM samples were incubated with PAMAM-PEG-Tf/TMZ for 24 h (Figure 2B). PAMAM-PEG-Tf/TMZ was indicated by red fluorescence staining, disrupted DNA was stained by green fluorescence and cell nuclei were visualized by DAPI counter-staining. As shown in Figure 2C, few TUNEL-positive cells appeared in the control group with no treatment, and an increased number of TUNEL-positive cells were observed when the sample was incubated with PAMAM-PEG-Tf/TMZ, PAMAM/TMZ or TMZ as compared the control (*P* < 0.01). There was no significant difference among the number of TUNEL-positive cells when the sample was incubated with PAMAM-PEG-Tf/TMZ, PAMAM/TMZ or TMZ (*P* > 0.05).

### Identification of sorted GSCs and non-GSCs

**Figure 3:**
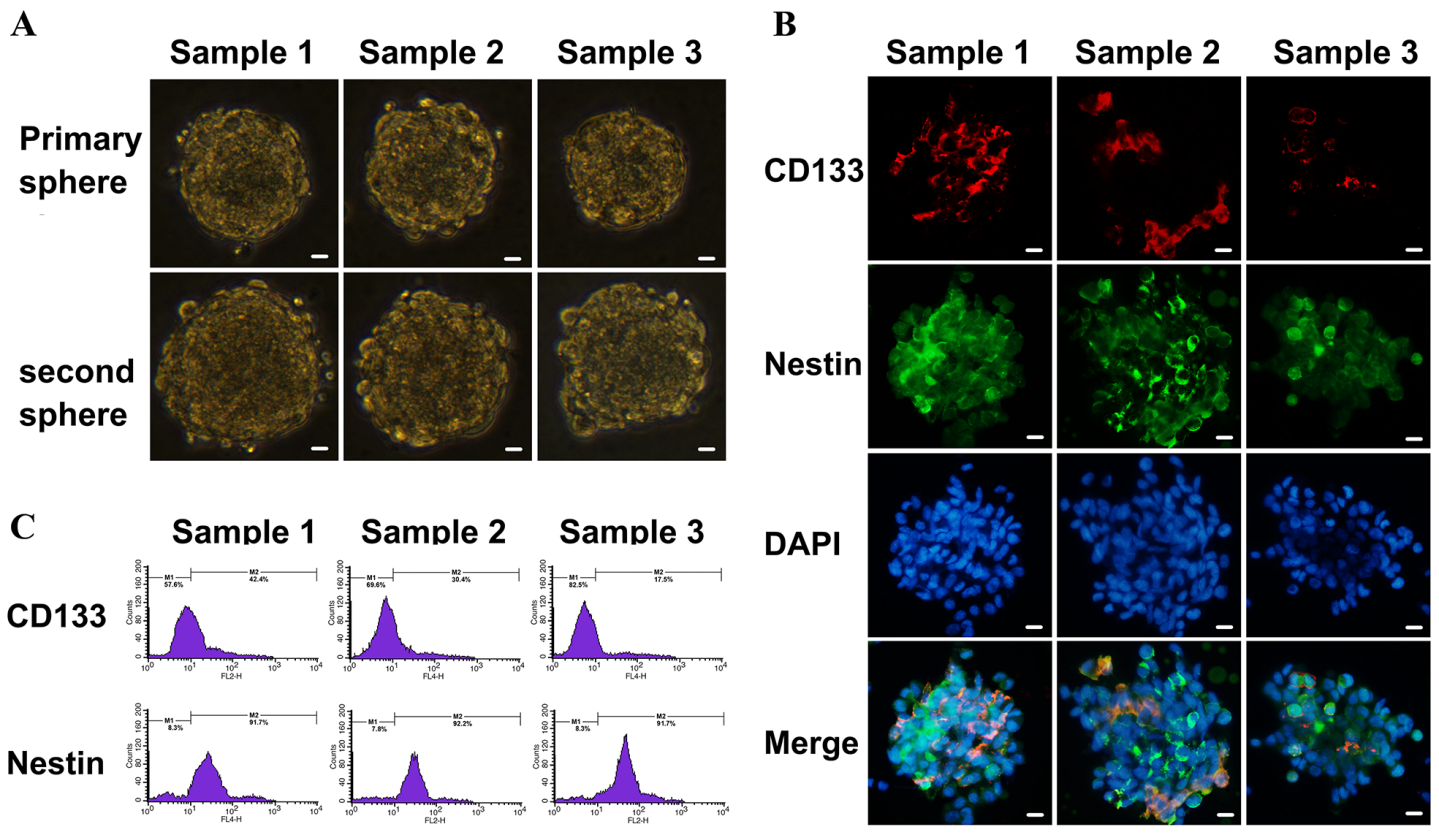
Identification of primary cultured GSCs from GBM samples Scale bar =10 μm. **(A)** Sub-sphere formation was confirmed after primary spheres were dissociated into single cells (×400 magnification). **(B)** The expressions of GSCs markers were detected using immunofluorescence with antibodies against CD133 (red fluorescence) and nestin (green fluorescence) to assess biological characteristic of cultured cells (×400 magnification). **(C)** The percentages of CD133^+^ and nestin^+^ cells in GSCs were analyzed by flow cytometry.

**Figure 4:**
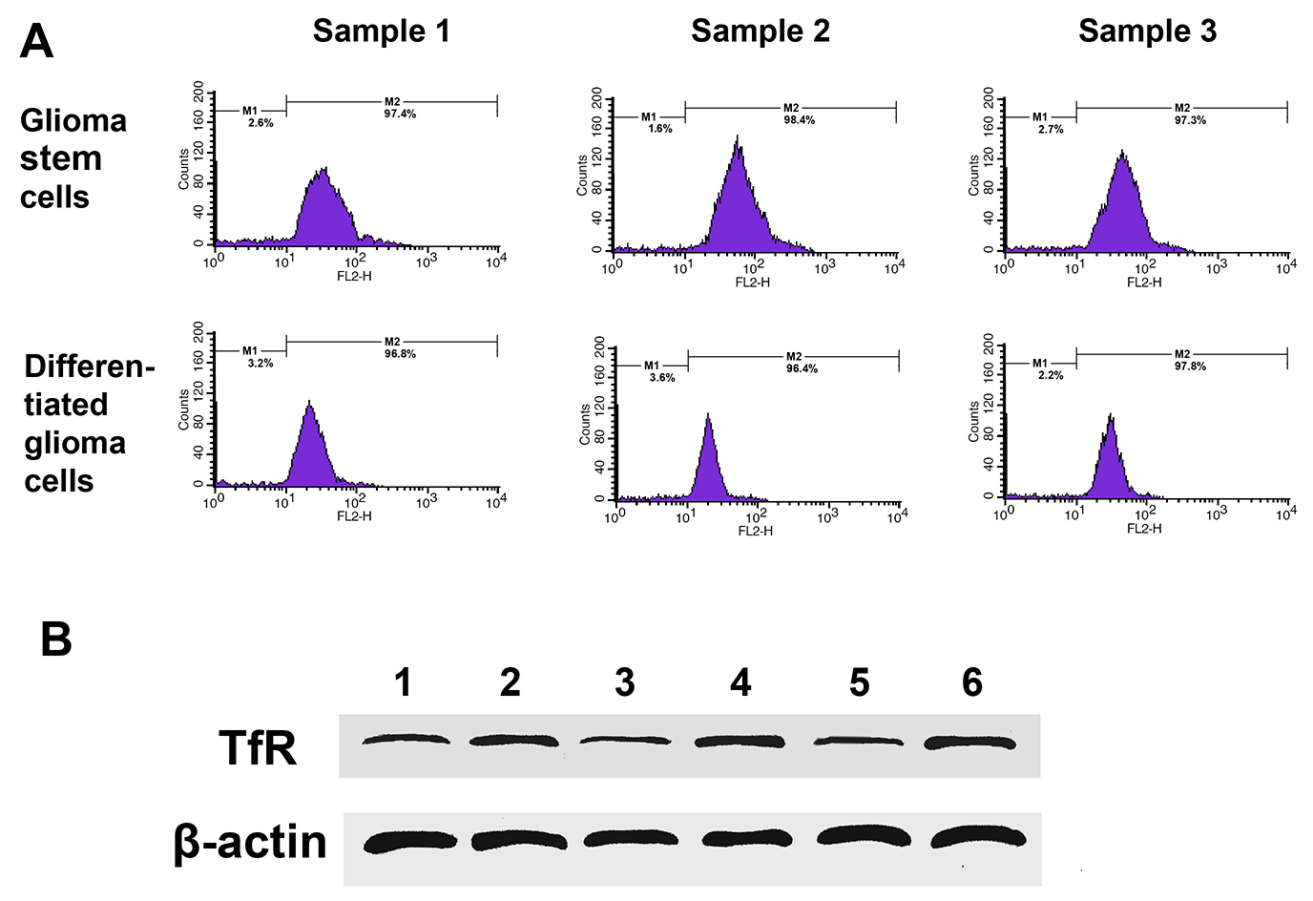
TfR expression in cultured cells from GBM resection samples **(A)** Flow cytometry analyzed TfR-positive cells number in GSCs and differentiated glioma cells. **(B)** Western blotting detected TfR expression. Lane 1, sample 1 differentiated glioma cells; Lane 2, sample 1 GSCs; Lane 3, sample 2 differentiated glioma cells; Lane 4, sample 2 GSCs; Lane 5, sample 3 differentiated glioma cells; Lane 6, sample 3 GSCs.

Isolated GSCs were identified by sub-sphere formation assay (Figure 3A) and surface marker analysis (Figure 3B). Tumor spheres were formed 3 to 4 weeks after primary culture of tumor cells from GBM patients. Subspheres were formed 4-5 days after primary spheres were dissociated. It was found that 100 ± 50 cells assembled in each subsphere, which was similar to that seen for primary spheres. GSCs showed strong expression of nestin and moderate expression of CD133 by immunofluoresence staining. Flow cytometry showed that (41.8 ± 1.4)%, (31.1 ± 2.8)% and (19.3 ± 1.5)% GSCs from surgical samples exhibited positive expression of the stem cell marker CD133, and at the same time (92.1 ± 2.9)%, (92.5 ± 5.7)% and (90.8 ± 3.9)% GSCs expressed positive nestin antigen, respectively (Figure 3C). The expression of TfR was higher in GSCs comparing to non-GSCs in the results of western blot (Figure 4A), and flow cytometry showed (97.9 ± 3.4)%, (98.2 ± 2.8)%, (97.8 ± 9.1)% TfR+ positive GSCs and (96.3 ± 2.8)%, (98.5 ± 4.4)%, (97.9 ± 3.2)% TfR+ positive non-GSCs (Figure 4B).

### Uptake and cytotoxicity of PAMAM-PEG-Tf/TMZ *in vitro*

**Figure 5:**
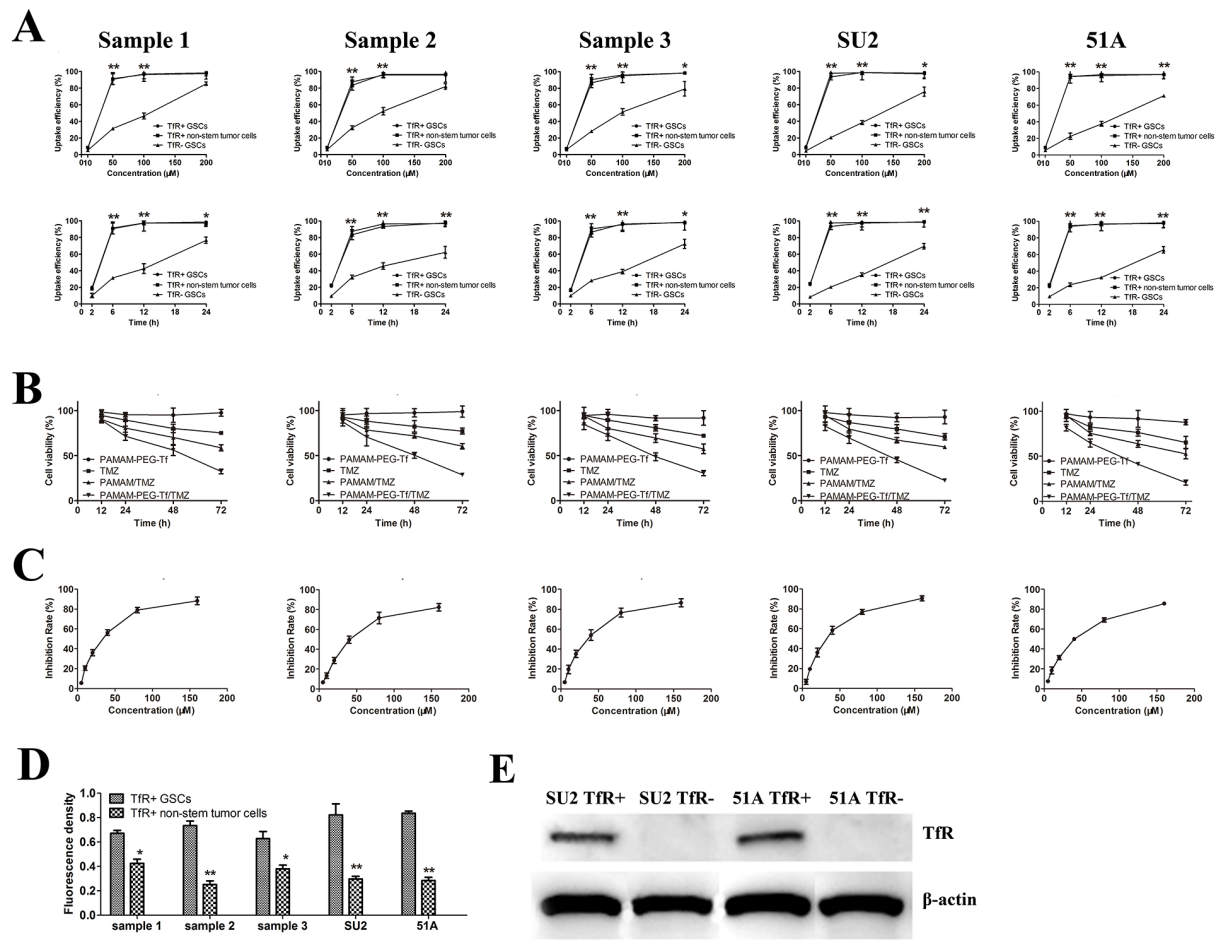
Uptake efficacy and proliferative suppression of PAMAM-PEG-Tf/TMZ *in vitro* **(A)** Uptake efficiency of cells were calculated after treatment with PAMAM-PEG-Tf/TMZ at different concentrations of TMZ ranging from 10 to 200 μM for 6 h or 50 μM for different intervals ranging from 2 to 24 h. *P < 0.05, **P < 0.01 vs. TfR- GSCs with the same concentration or at the same period; **(B)** PAMAM-PEG-Tf/TMZ inhibited the proliferation of TfR+ GSCs at 12, 24, 48 and 72 h after treatment with 50 μM TMZ, PAMAM/TMZ and PAMAM-PEG-Tf/TMZ. *P < 0.05, **P < 0.01 vs. TMZ; **(C)** Growth curves of cells were painted when treatment with different concentration PAMAM-PEG-Tf/TMZ. **(D)** Fluorescent intensities of TfR+ GSCs and non-stem tumor cells were analyzed when treatment with 50 μM PAMAM-PEG-Tf/TMZ at 6 h. *P < 0.05, **P < 0.01 vs. TfR^+^ GSCs. **(E)** Expressive levels of TfR were validated using western blot in TfR- GSCs, which were knocked down by RNAi method.

Cells were observed under the fluorescence microscope following time and dose-dependent incubation with PAMAM-PEG-Tf/TMZ with the aim of determining uptake efficiency. As shown in Figure 5A, uptake efficiency was no different up to two hours later when PAMAM-PEG-Tf/TMZ (50 μM TMZ) was added to TfR+ cells as compared TfR- cells (*P* > 0.05). However, significant increases in uptake efficiency occurred following 6 h incubation in TfR+ cells from all samples as compared TfR- cells (*P* < 0.01). Uptake efficiencies of free Tf by TfR+ Sample 1, 2, 3, SU2 and 51A GSCs were (96.6 ± 6.8)%, (93.8 ± 3.1)%, (95.4 ± 7.6)%, (98.2 ± 5.5)% and (92.4 ± 8.7)%, respectively , after 6 h free Tf incubation.

We examined the effects of PAMAM-PEG-Tf/TMZ on cell proliferation of TfR+ GSCs *in vitro* using an MTT assay. Cell viability was assessed after treatment with 50μM TMZ, PAMAM/TMZ, PAMAM-PEG-Tf/TMZ and PAMAM-PEG-Tf for 12, 24, 48 and 72 h (Figure 5B). Treatment with PAMAM-PEG-Tf/TMZ inhibited proliferation of TfR+ GSCs time-dependently. Cell viability of TfR+ GSCs from samples 1, 2 and 3 were (56.2 ± 2.1%), (50.4 ± 3.3%) and (48.7 ± 4.3%), respectively when treated with PAMAM-PEG-Tf/TMZ for 48 h; viabilities that had decreased significantly as compared with the TMZ and PAMAM/TMZ groups (*P* < 0.01). Growth curves were showed in Figure 5C when cells were treated in PAMAM-PEG-Tf/TMZ of different concentration for 48 h, and the value of half maximal inhibitory concentration (IC50) of PAMAM-PEG-Tf/TMZ was showed in Table 1.

**Table 1:** IC50 of PAMAM-PEG-Tf/TMZ for GSCs

Cells	IC50 (μM)	95% Confidence interval
Sample 1	31.69	27.33-36.81
Sample 2	41.57	35.49-49.10
Sample 3	33.53	28.75-39.24
SU2	31.03	26.80-35.99
51A	38.27	32.49-15.42

Fluorescence densities from PAMAM-PEG-Tf/TMZ in TfR+ GSCs as compared matched non-stem stem cells was markedly higher (*P* < 0.05 or *P* < 0.01; Figure 5D), and western blot analysis showed no expression of TfR on TfR- SU2 and 51A cells, which indicated successful TfR knockdown using siRNA (Figure 5E).

### BBB permeability in mouse brain with xenografts

**Figure 6:**
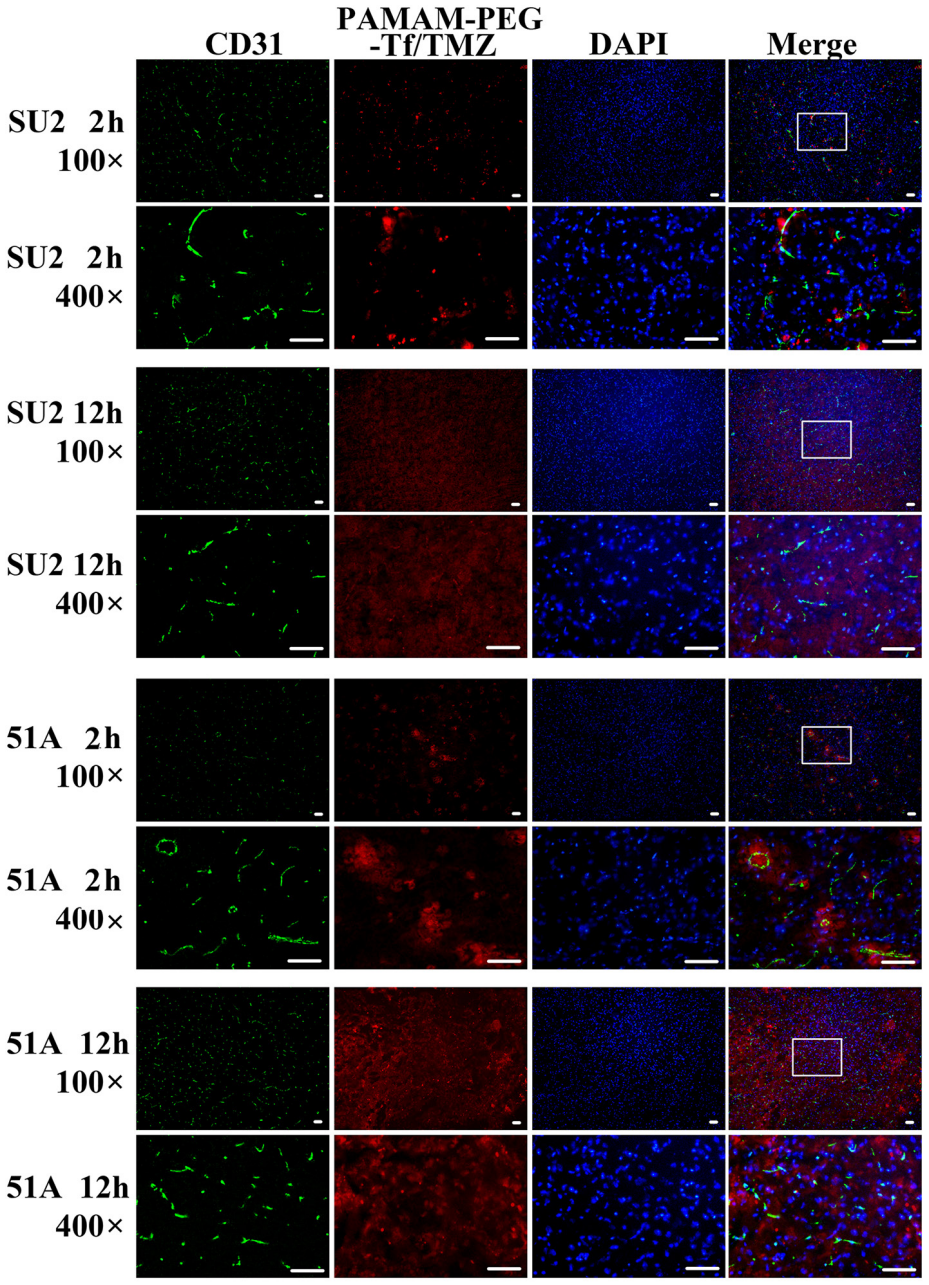
BBB permeability of PAMAM-PEG-Tf/TMZ in tumor-bearing mice Murine tumors that had been intracranially implanted with GSCs were used for the study of BBB permeability at periods of 2 h and 12 h post-intravascular adoptive transfer. Red signal patterns were derived from PAMAM-PEG-Tf/TMZ, and green signal patterns were derived from immunostaining anti-CD31 antibody. The fluorescent images at a magnification of ×100, were shown in the white pane, which was magnified to a ×400 field view, and shown on the next line. Scale bar =50 μm

Analysis on PAMAM-PEG-Tf/TMZ permeability to the BBB was performed at 2 h and 12h post-intravascular injection (Figure 6). Images from mouse brain tumors were obtained, and microvessels were shown by a green signal from CD31 stained cells. Red signals from PAMAM-PEG-Tf/TMZ conjugated with the Alexa Fluor 555 unit from TfR, were seen near blood vessels 2 h post-administration. The red signal of the entire imaged area in the mouse brain tumor was shown on images 12 h post-administration.

### The study of cytotoxicity and uptake in SOX2+ cells from intracranial xenografts

**Figure 7:**
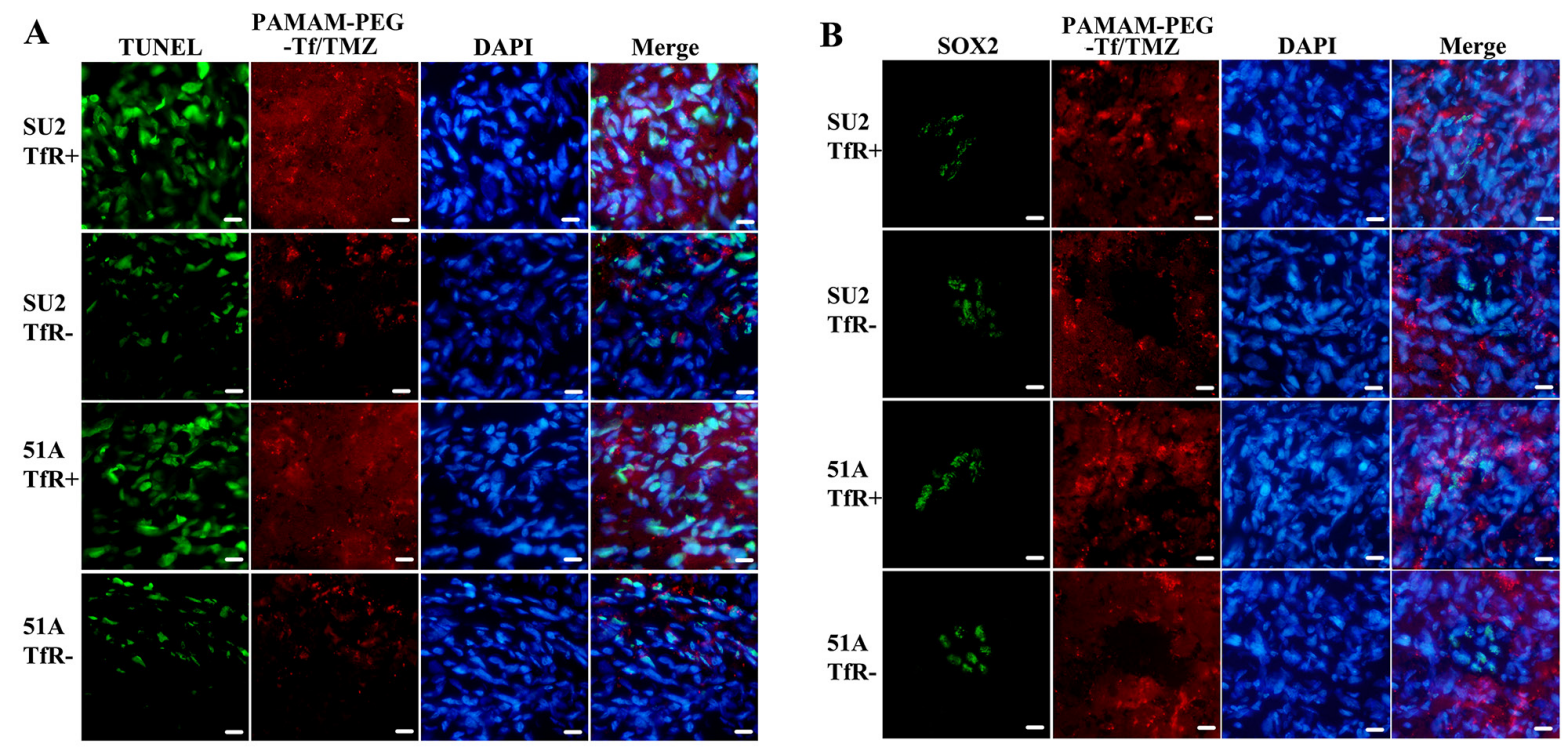
Uptake and cytotoxicity of PAMAM-PEG-Tf/TMZ in nude BALB/c mice bearing the TfR+/- tumor cells Intracranial xenografts from GSCs were used to evaluate uptake and cytotoxicity after 24 h following intravascular administration with 5 mg/kg TMZ. Red signal patterns were derived from PAMAM-PEG-Tf/TMZ. **(A)** Cytotoxicity assay of PAMAM-PEG-Tf/TMZ. Disrupted DNA was stained by green fluorescence using the TUNEL assay in the apoptotic study. **(B)** Detection of the uptake of PAMAM-PEG-Tf/TMZ by stem cells. The uptake of PAMAM-PEG-Tf/TMZ by SOX2+ cells was shown by SOX2 immunostaining using green fluorescence. Scale bar =10 μm.

Cellular apoptosis from murine brain tumors was evaluated by TUNEL staining after 24 h of treatment with PAMAM-PEG-Tf/TMZ, in which, disrupted DNA showed a green signal (Figure 7A). A number of apoptotic cells appeared in the area of the red signal from Tf with Alexa Fluor 555, which indicated active apoptosis in tumor cells absorbing to PAMAM-PEG-Tf/TMZ. The uptake by brain tumors and location in SOX2+ cells of PAMAM-PEG-Tf/TMZ are shown in Figure 7B 24 h after tumor-bearing animals were injected. The percentage of apoptotic cells in TfR+ SU2 (63.0 ± 4.2)% and TfR+ 51A (71.1 ± 4.9)% xenografts was increased significantly than that in TfR- SU2 (29.1 ± 2.6)% and (24.5 ± 3.1)% (*P* < 0.01).

Microscopic examination of the brains revealed targeting features of PAMAM-PEG-Tf/TMZ in the tumor. PAMAM-PEG-Tf/TMZ was indicated by red fluorescence, and SOX2+ cells were shown by green fluorescence by immunostaining analysis. Xenografts from TfR+ cells showed a comprehensive red fluorescence, and SOX2+ cells similarly showed full patterns of red fluorescence as compared TfR- cell xenografts. In addition, minimal red fluorescence was seen in SOX2+ cells in spite of a complete moderate intensity of red fluorescence in SOX- cells. As determined by fluorescence of individual mice, PAMAM-PEG-Tf/TMZ was absorbed by SOX2+/TfR+ cells, but not by SOX2+/TfR- cells. This observation indicated a specific location in TfR+ GSCs *in vivo*.

### Therapeutic efficacy *in vivo*

**Figure 8:**
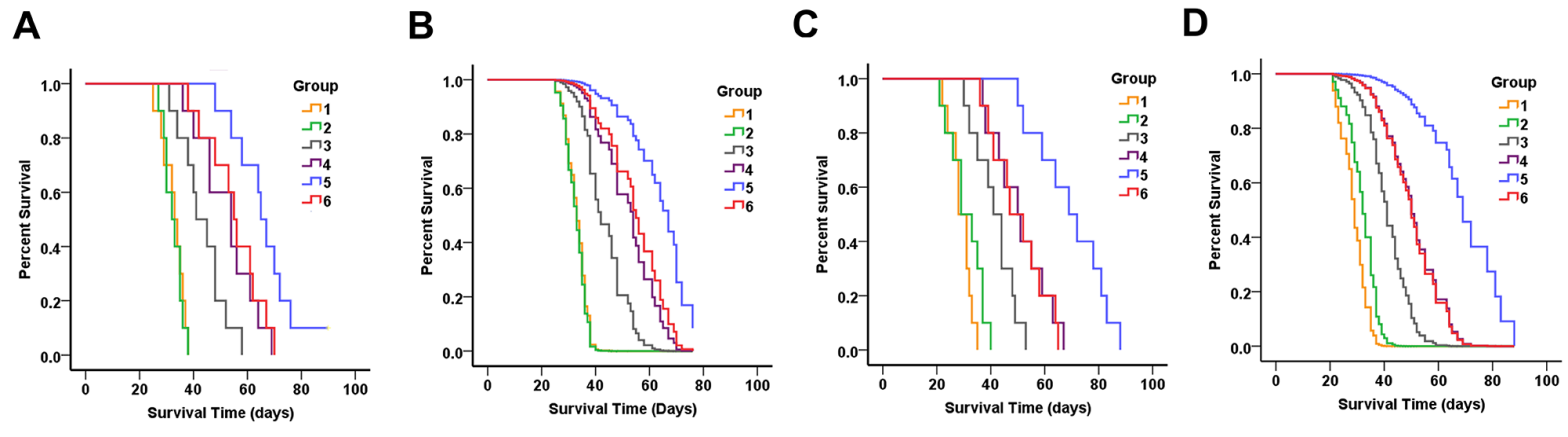
Therapeutic efficacy of PAMAM-PEG-Tf/TMZ in nude BALB/c mice bearing intracranial SU2 (A for Kaplan-Meier curves and B for Cox survival plots) and 51A (C for Kaplan-Meier curves and D for Cox survival plots) cellular xenografts Animals were intravenously treated with normal saline (group 1); intravenously administered PAMAM-PEG-Tf (group 2); orally administered TMZ (group 3); intravenously administered PAMAM/TMZ (group 4); intravenously administered PAMAM-PEG-TfR/TMZ for TfR+ glioma xenografts (group 5); and intravenously administered PAMAM-PEG-TfR/TMZ for TfR- glioma xenografts (group 6). Drugs were administered continuously once per day for a total period of 5 days after intracranial tumor formation. A longer period of survival was shown in mice that exhibited TfR+ glioma xenografts when intravenously administered with PAMAM-PEG-TfR/TMZ as compared to controls, PAMAM-PEG-Tf, TMZ alone, PAMAM/TMZ and PAMAM-PEG-TfR/TMZ for TfR- glioma xenografts.

The efficacy of oral TMZ and i.v. TMZ nanoparticles for tumor suppression was evaluated in the nude mouse intracranial xenograft models. The status of the brain tumor and suppression of tumor growth were monitored since mice were treated by chemotherapy. Figure 8 and Table 2 showed survival results using Kaplan-Meier curves, and multiple group comparisons were described using Cox survival plots. Significant anti-tumor efficacy was observed in all treated groups. For SU2 cells implanting mice (Figure 8A), the median survival time (MST) of the mice administered oral TMZ was 43.5 ± 8.3 days (95% CI, 37.6 – 49.4 days), and exhibited significant antitumor effects as compared the control group 32.6 ± 4.1 days (95% CI, 29.6 – 35.6 days; *P* < 0.01) and i.v. PAMAM-PEG-Tf group 32.5 ± 3.5 days (95% CI, 30.0 – 35.0 days; *P* < 0.01). There was no statistical difference between MST of control and PAMAM-PEG-Tf group (*P* > 0.05). Moreover, MSTs of mice with TfR+ cellular xenografts for PAMAM/TMZ treatment and in TfR- cellular xenografts for PAMAM-PEG-TfR/TMZ treatment were 52.6 ± 10.6 days (95% CI, 45.0 – 60.2 days) and 55.2 ± 10.4 days (95% CI, 47.8 – 62.6 days), which showed marked statistical differences on comparing TMZ administration (*P* < 0.05).

**Table 2:** Survival times of mice transplanted with GSCs xenografts following treatment (d)

Cells	Group	Treatment	Range	Mean±SE	Median
SU2	1	untreated control for TfR+ cells	25-38	32.6 ± 4.1	33
	2	PAMAM-PEG-Tf for TfR+cells	27-28	32.5 ± 3.5	32
	3	TMZ for TfR+cells	31-58	43.5 ± 8.3**^##^	41
	4	PAMAM/TMZ for TfR+cells	36-69	52.6 ± 10.6^$^	54
	5	PAMAM-PEG-TfR/TMZ for TfR+ cells	48->90	66.4 ± 11.9^%%&&^	65
	6	PAMAM-PEG-TfR/TMZ for TfR- cells	38-70	55.2 ± 10.4^$^	55
51A	1	untreated control for TfR+ cells	22-35	29.1 ± 4.1	28
	2	PAMAM-PEG-Tf for TfR+cells	21-40	31.0 ± 6.4	29
	3	TMZ for TfR+cells	30-53	41.5 ± 7.6**^#^	41
	4	PAMAM/TMZ for TfR+cells	37-67	50.8 ± 10.3^$^	50
	5	PAMAM-PEG-TfR/TMZ for TfR+ cells	50-88	69.6 ± 13.2^%%&&^	69
	6	PAMAM-PEG-TfR/TMZ for TfR- cells	36-65	50.3 ± 10.2^$^	47

10 mice were included in each treatment group, sacrificed when symptoms of death appeared, and survival times were calculated according to the time between tumor transplantation and sacrifice. There was one mice surviving more than 90 days in group 5 in SU2 cells. ^**^P < 0.01 vs. group 1; ^#^*P* < 0.05, ^##^*P* < 0.01 vs. group 2; ^$^*P* < 0.05 vs. group 3; ^%^*P* < 0.05, ^%%^*P* < 0.01 vs. group 4; ^&&^*P* < 0.05, ^&&^*P* < 0.01 vs. group 6.

Mice bearing TfR+ gliomas and that had received i.v. PAMAM-PEG-TfR/TMZ, also showed an MST of 66.4 ± 11.9 days (95% CI, 57.9 – 74.9 days), which was significantly longer as compared to that of TfR-cell implanted mice and PAMAM/TMZ administration (*P* < 0.01). PAMAM-PEG-Tf/TMZ administration retarded xenograft growth from TfR+ cells, and did so most significantly among the treated groups. Survival data of 51A cells (Figure 8C) that had been implanted in mice was accordant with that of SU2 cells. When receiving the PAMAM-PEG-TfR/TMZ injection, MST of mice bearing TfR+ 51A cells was 69.6 ± 13.2 days, which was significantly increased as compared control (29.1 ± 4.1 days), PAMAM-PEG-Tf administration (31.0 ± 6.4 days), TMZ administration (41.5 ± 7.6 days), PAMAM/TMZ treatment (50.8 ± 10.3 days) and PAMAM-PEG-TfR/TMZ treatment for TfR- 51A implanted mice (50.3 ± 10.2 days).

Results indicate that when administrated with TMZ, the survival time both in SU2 and 51A cells implanted mice was significantly different from that of counterpart control group (*P* < 0.01). The difference in MSTs between groups of mice administrated with PAMAM-PEG-TfR/TMZ for TfR- cells and PAMAM/TMZ administration for TfR+ cells were not significant (*P* > 0.05). Non-targeting nanoparticles encapsulating TMZ extended survival time of GSCs implanted mice comparing to TMZ treatment alone (*P* < 0.05). Statistically significant differences were noted in TfR targeting and non-targeting groups of nanoparticles encapsulating TMZ (*P* < 0.01). Multivariate analysis and Cox proportional hazards model indicated that TMZ, nanoparticle and targeting factor correlated significantly with survival in mice bearing brain tumors (Figure 8B for SU2 and Figure 8D for 51A cells implanted mice).

## DISCUSSION

In this study we evaluated the validity of PAMAM-PEG-Tf/TMZ nanoparticles in damaging GSCs, as well as non-stem tumor cells. Nanoparticles that were targeted to TfR, with high expression in both populations, were prepared by conjugating Tf to PAMAM dendrimers, which could specifically target TMZ to brain tumors, and especially GSCs.

Overexpressed TfR on active proliferating surfaces of tumor cells is widely used to deliver drugs since iron is a basic element that is required during cellular metabolism. Complexes taking TfR as a target, is produced by Mebiopharm, SynerGene Therapeutics and Calando Pharmaceuticals, and has been used in clinical trials to deliver anti-cancer drugs [10].

TfR targeting is a sufficient anti-cancer therapy, so much so that a common marker is used to target both GSCs and non-stem tumor cell populations. However, taking TfR as a target to delete glioma is a more practical strategy due to over-expression on the surfaces of both populations. In previous studies, the prepared nanoparticles, which comprised a liposomal complex employing an anti-TfR single-chain variable fragment as a targeting ligand, carried the wtp53 gene, and showed anti-cancer activity by inducing death of both cancer stem cells and non-stem cancer cells [11]. This approach was used in Phase I trials in advanced solid cancer patients [12, 13]. Combined use of nanoparticles and conditional TMZ chemotherapy increased its tumoricidal efficacy in TMZ-resistant glioma transplanted mice [14].

This study demonstrated that PAMAM-PEG-TfR/TMZ not only inhibited glioma growth, but also accomplished tumor regression and delayed tumor recurrence, at least in orthotopic glioma nude mouse models. Such responses were likely produced by elimination of both GSCs and non-stem tumor cells, which indicated the broad applicability of targeting both populations. The specificity of PAMAM-PEG-Tf/TMZ also prevented significant side-effects of TMZ because of the TfR targeting effect. High uptake efficiency was observed by both populations *in vitro* with similar frequencies; however, a more significant fluorescence intensity was shown in GSCs as compared non-stem tumor cells at 12 h after 50μM PAMAM-PEG-Tf/TMZ treatment. The targeting ability of PAMAM-PEG-Tf/TMZ was further confirmed *in vivo* using intracranial transplanted tumor nude mice. Red fluorescence from PAMAM-PEG-Tf/TMZ was readily seen in the tumor area.

The fluorescent images showed clearly that the PAMAM-PEG-Tf/TMZ and the stem cell marker SOX2, were co-localized in intracranial xenografts of TfR+ GSCs, and evident apoptosis was present in the cells that fully exhibited PAMAM-PEG-Tf/TMZ. No absorption of PAMAM-PEG-Tf/TMZ was seen in SOX2+ cells from TfR- GSC xenografts. Therefore, PAMAM-PEG-Tf/TMZ suppressed tumor growth more effectively than TMZ and PAMAM/TMZ due to drug accumulation by TfR+ GSCs.

MSTs of mice with the TfR+ SU2 or 51A xenograft that was treated with PAMAM/TMZ, was similar to that as its TfR- counterpart of SU2 or the 51A xenograft, which received PAMAM-PEG-Tf/TMZ, due to no TfR targeting. However, the anti-tumor efficacy of nanoparticles comprised of PAMAM/TMZ and PAMAM-PEG-Tf/TMZ, was more potent than that of free TMZ. Due to non-targeting nanoparticle uptake by cells, PAMAM-PEG-Tf/TMZ was absorbed more easily by TfR- non-stem tumor cells due in part to the decreased dose, and longer period of therapy as compared with the targeting particles. Moreover, TfR- GSCs failed to absorb PAMAM-PEG-Tf/TMZ, but TfR- non-stem tumor cells could absorb abundant drugs, which indicated that the prepared nanoparticles were not absorbed by GSCs. Thus, targeting GSCs is an important option in preventing glioma recurrence.

Drug nanocarriers should need the characteristics of high drug loading and entrapment rate, biodegradable carrier material, low or no toxicity, appropriate particle size and long cycle period. The structure characteristics of common drug nanocarrier are showed in Table 3. Non-targeting uptake is obvious obstacle of small molecular chemical drugs for cancer therapy. A large amount of experimental researches focus on further development of novel and more efficient delivery systems. Nanoparticles linked with a ligand are widely used for the delivery of anticancer drugs. Application and synthesis of delivery vehicles targeted TfR played an important role because the TFR is overexpressed on the surface of various fast-growing malignant tumor cells. Receptor-mediated endocytosis of TF induced rapid tumor-cell-specific uptake of targeting nanoparticles, and the internalized nanoparticles could be effectively degraded to release functional drugs molecules in the cells. Kanwar et al., prepared iron-saturated bovine lactoferrin, and displayed a 60-80% similar sequence with Tf nanocapsules, an observation that validated the capacity of these nano-capsules to kill colon cancer stem cells and induce apoptosis by targeting survivin [15]. The transferrin-targeting nanoparticle delivery system that carries the tumor suppressor microRNA-1, efficiently delivered miR-1 and inhibited migration of GBM patient-derived GSC-enriched spheres [16]. Nanocomplexes of cationic liposomes that were conjugated with TfR single-chain antibody fragments and which encapsulated TMZ were taken up by cancer stem cells [17]. Concordant with our results, these previous studies all showed ligands of TfR that had the ability to target cancer stem cells.

**Table 3:** The structure characteristics of common drug nanocarrier

Nanocarriers	Structure
Nanoparticles	Nanoparticles are composed of polymer material capsules and liquid (water or oil) inner cores. The drug is usually encapsulated by the polymer film in the inner core layer. Nanoparticles include polylactic acid (PLA), polyglycolic acid (PGA), polycaprolactone (PCL), polylactic acid glycolic acid (PLGA), natural polymer materials, and so on.
Nano-liposomes	Nano-liposomes are multi-layer vesicle structure formed by phospholipids. They are. each layer are composed of lipid bimolecular membrane, interlayer and liposome inner core are the water phase, and the bimolecular membrane is oil phase.
Nano-micelles	Nano-micelles are composed of hydrophilic shell and hydrophobic core. Hydrophilic segments include polyethylene glycol (PEG), polyethylene oxide (PEO), polyoxypropylene, and so on, and hydrophobic segments include PLA, PGA,PCL,PLGA, Chitosan, and so on.
Nano-magnetic particles	Nano-magnetic particles are composed of drug magnet particle carrier and skeleton material.
Dendrimers	Dendrimers are symmetrically spherical polymer, showing a dendritic geometric appearance. Their molecular surfaces have functional groups with very high density, and wide cavities exist inside the molecules.

Temozolomide (TMZ) was approved by the FDA as one of the most commonly used alkylating agents to target glioblastoma. MGMT promoter methylation as compared un-methylation is associated with longer survival when GBM patients are treated with TMZ [18]. DNA methylation signatures from primary cultured glioma cells were present in xenograft tumors, which indicated no tissue culture-related epigenetic changes [19]. This study selectively cultured GSCs from clinical samples or cell-lines that possessed the methylated MGMT promoter. These GSCs were sensitive to TMZ treatment, and could be used to validate the anti-tumor efficacy of PAMAM-PEG-Tf/TMZ. PAMAM-PEG-Tf/TMZ solution when administered intravenously to experimental mice with the aim of examining its anti-cancer efficacy. The results mimicked *in vitro* experiments and were concordant with observations made for clinical samples. Cytotoxicity was observed for *in vitro* cultured CSCs and non-stem tumor cells. Moreover, apoptosis was shown following *in vivo* administration and in studies of clinical samples.

Co-localization of CD31 positive cell staining and PAMAM-PEG-Tf/TMZ was seen to have accumulated in the xenograft. PAMAM-PEG-Tf/TMZ accumulated from nearby vessels two hours post-administration to a wide area including far from the vessels at 12 h post-administration. This observation indicated enhanced permeability of the BBB. TfR-targeting nanoparticles could efficiently deliver drugs through the BBB. In previous studies, and due to its ability to cross the BBB, Tf-targeted nanoparticles incorporating zoledronic acid increased the tumoricidal efficacy of this drug in intracranial U373 xenografts [20]. The delivery of a Tf-conjugated magnetic silica nanoparticle complex that was loaded with doxorubicin and paclitaxel, was enhanced in the intracranial U87 xenograft of BALB/c nude mice[21]. Our results were concordant with these previously published studies of nanoparticles that used Tf as the targeting ligand, which was shown to quite easily penetrate BBB.

## CONCLUSION

Overall, in this study, the efficacy of a novel nanoparticle complex of PAMAM-PEG-Tf/TMZ was evaluated to GSCs. A high dose uptake and significant cytotoxicity of PAMAM-PEG-Tf/TMZ in GSCs was observed due to the targeting function of Tf, in which the ligand was highly expressed in GSCs. PAMAM-PEG-Tf/TMZ traversed the BBB and delivered TMZ to the avascular region of tumor, and delivered an effective dose of TMZ specifically to tumor cells. After surgery and radiotherapy, the chemotherapeutic protocol was commonly ineffective against drug-resistant GSCs. The achieved delivery mechanism, provoked a potent induction of glioma cell apoptosis, and especially GSCs. The targeting TfR nanoparticles could be used for an effective therapeutic strategy against GSCs, and against non-stem tumor cells, and it would provide a promising tumoricidal strategy for treating glioma displaying MGMT promoter methylation.

## MATERIALS AND METHODS

### Synthesis of PAMAM-PEG–Tf bioconjugates

**Figure 9:**
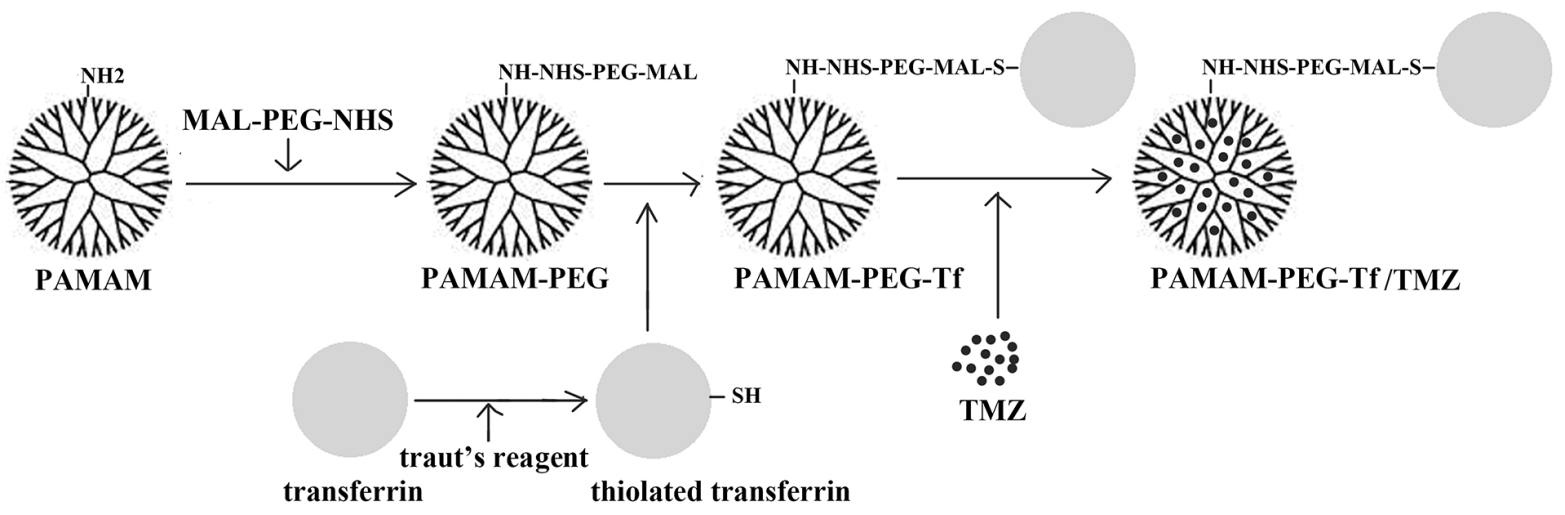
Preparation of Tf targeting nanoparticle A Tf was thiolated by traut’s reagent to yield a thiolated Tf. PAMAM dendrimer was conjugated to MAL group of MAL-PEG-HNS (PAMA-PEG), then HNS group was conjugated with thiolated Tf to form PAMAM-PEG-Tf. TMZ was encapsuled in the cavities of PAMAM-PEG-Tf.

PAMAM and Tf were conjugated by bifunctional groups of maleimide-polyethylene glycol 2000-amino succinimidyl succinate (MAL-PEG2000-NHS) to synthesize PAMAM-PEG-Tf bioconjugates (Figure 9). PAMAM dendrimers were reacted with MAL-PEG2000-NHS at a ratio of 1:10 (mol/mol) in PBS (pH 8.0) for 2 h at room temperature. The surface NH_2_ groups of PAMAM were specifically reacted with the NHS groups of MAL-PEG2000-NHS. The resulting conjugate, PAMAM-PEG, was purified by ultrafiltration through a molecular weight cut-off membrane of 5KDa, following which, the buffer was changed to PBS pH7.0. Simultaneously, Tf with an Alexa Fluor 555 (Molecular Probes) fluorochrome was thiolated using Traut’s reagent, and then the thiolated Tf was coupled to the periphery of PAMAM-PEG at a ratio of 1:5 (PAMAM to peptide, mol/mol) in PBS pH 7.0 for 24 h at room temperature. The MAL groups of PAMAM-PEG were specifically reacted with the thiol groups of thiolated Tf. To purify the bioconjugated product, gel filtration was employed with a Sephacryl S-300 gel filtration column. To examine TfR conjugation and PAMAM, the absorbance was measured at a wavelength of 280 nm, and bioconjugated synthesis was determined by ultraviolet-visible spectra, and the purity of bioconjugate synthesis was characterized by SDS-PAGE that was stained with Coomassie Brilliant Blue. Protein concentrations of synthesized bioconjugates were determined by measuring absorbance at 280 nm using a multiskan spectrophotometer model 1510 (Thermo Fisher Scientific, Vantaa, Finland) according to a free Tf standard curve.

### Preparation of PAMAM-PEG –Tf/TMZ nanoparticles

TMZ was encapsulated in the interior of the PAMAM-PEG-Tf by mixing TMZ with the carriers. Next, the free TMZ molecules were separated by gel filtration chromatography. The relative dose of TMZ in PAMAM and PAMAM-PEG-Tf was calculated using the TMZ standard curve with a maximum absorption of 329nm. The encapsulating efficiency (EE%) and the drug loading capacity (LC%) of TMZ in PAMAM and PAMAM-PEG-Tf was determined by measuring TMZ concentration. Both EE% and LC% were calculated as indicated below. EE% = (TMZ in PAMAM or PAMAM-PEG-Tf / total amount of TMZ in solution) ×100%, LC% = (TMZ in PAMAM or PAMAM-PEG-Tf / solutes total weight) ×100%. Preparation of PAMAM-PEG-Tf /TMZ was determined by ultraviolet-visible spectra.

### Statement of ethics

Tumor tissues from three pathologically diagnosed human GBM (Grade IV) surgical resections were used for immunohistochemistry, MSP and primary cell culture. The patients had provided their written and informed consent, which were under Institutional Review Board approval of the First Affiliated Hospital of Soochow University, China.

### DNA extraction and MSP

Genomic DNA was isolated from frozen GBM tissue (Qiagen, Germany), then bisulfite conversion was performed (ZYMO Research, USA). The methylation status of the MGMT genes was determined by a nested, two-stage PCR assay [19]. The Primer sequences and amplified fragments of PCR products were as follows:

Stage-1: MGMT-Forward, 5′-GGATATGTTGGGATAGTT-3’, MGMT-Reverse, 5′-CCAAAAACCCCAAACCC-3′ (289bp); and Stage-2: methylated MGMT Forward, 5′-TTTCGACGTTCGTAGGTTTTCGC-3’, methylated MGMT Reverse, 5′- GCACTCTTCCGAAAACGAAACG -3’ (81bp), unmethylated MGMT Forward, 5′- TTTGTGTTTTGATGTTTGTAGGTTTTTGT-3’, unmethylated MGMT Reverse, 5′- AACTCCACACTCTTCCAAAAACAAAACA-3’ (93bp). The PCR products were separated on two percent agarose gels.

### Uptake and cytotoxic assay in surgical samples of glioblastoma

GBM tissues from surgical excision procedures were collected immediately for the measurement of uptake and cytotoxicity assay as previously described [22]. The samples were incubated with PAMAM-PEG-Tf/TMZ (50 μM TMZ) for 24 h, washed twice and frozen embedded in an optimal cutting temperature (OCT) compound. Then 10 μm cryostat sections were prepared using an ultracryotome, following which, apoptotic cells in the GBM samples were detected by terminal deoxynucleotidyl transferase (TdT)-mediated dUTP-biotin nick end labeling (TUNEL) assay, using an In Situ Cell Death Detection Kit (Promega, USA) and used according to the manufacturer’s instructions. Sections were counter-stained with anti-fade sealant containing 4’6-diamidino-2-phenylindole (DAPI). Fluorescence images were visualized and captured using a fluorescence microscope (Olympus BX40, Japan).

### Primary cell culture of human brain tumors and cell-lines

Freshly resected human glioblastoma tumor samples were dissociated for primary cell culture. Tumor tissues were washed with phosphate buffered saline (PBS), minced mechanically into small fragments and dissociated into single cells and/or small clumps by tripsin.

Cells were cultured on non-adherent plates in serum-free DMEM/F12 medium containing 2%B27 supplement (Gibco, USA), 20 ng/ml epidermal growth factor (EGF, Invitrogen, USA) and 20 ng/ml basic fibroblast growth factor (bFGF, Invitrogen) to facilitate the growth of tumor spheres. Cells were cultured by changing half of the medium every 3 days. Primary neurospheres were dissociated into single cells and cultured for 5 days to determine the capacity to form secondary spheres.

SU2 cells are a stem cell-line of Chinese glioma origin, were originally isolated from a female patient with recurrent GBM, and provided as a gift from Professor Qiang Huang [23]. Also, 51A cells are a glioma stem cell-line from a recurrent GBM, and provided as a gift from Professor Yihong Zhou [24]. GSCs were cultured in DMEM/F12 medium that was supplemented with 10% fetal bovine serum to induce differentiation. All cells were cultured in a humidified atmosphere of 5% CO_2_ in air at 37°C.

### Immunofluorescence staining, western blot and flow cytometry analysis

Neurospheres that adhered to poly L-lysine-coated slides were used for studies of surface markers. Primary antibodies used included mouse anti-nestin (1:250, Abcam) and rabbit anti-CD133 (1:5, Miltenyi Biotec GmbH). Secondary antibodies were anti-mouse Alexa Fluor 488 and anti-rabbit Alexa Fluor 555 (Molecular Probes, Eugene, OR, USA). Images were captured by fluorescence microscope.

Neurospheres were dissociated into single cell suspensions, and then incubated with PE-conjugated nestin (1:100; Miltenyi Biotech GmbH) and APC-conjugated CD133 (1:100; Miltenyi Biotech GmbH) antibody. Mouse anti-TfR primary antibody (1:200, Invitrogen) and PE-conjugated mouse IgG (1:100, Invitrogen) were used. Labeled cells were analyzed by flow cytometry (Beckton Dickinson FACScan; BD Biosciences). Cells were collected and lysed for western blotting analysis by standard procedures. Primary antibodies of anti-TfR (1:1000) and anti-β-actin (Sigma,USA) were used.

### Generation of stable TfR-negative GSCs

Cells were transfected with siRNA to suppress TfR gene expression using a lentiviral-mediated gene transfection kit (Shanghai GenePharma Co.Ltd., China) according to the manufacturer’s instructions. Cells were used experimentally according to a transfection efficiency of more than 85 per cent. GSCs (TfR+ GSCs) and GSCs that were transfected with the siRNA-TfR vector (TfR- GSCs) were assayed by western blotting.

### Uptake and cytotoxicity of PAMAM-PEG-Tf/TMZ *in vitro*

Cells were suspended in 2 mL of nutrient mediumat a density of 2×10^5^ and seeded in 6-well plates for 24 h. TfR+ and TfR- cells were dose- and time-dependently incubated with PAMAM-PEG-Tf/TMZ. The fluorescent images that were obtained post-incubation at an excitation wavelength of 555 nm showed uptake of PAMAM-PEG-Tf/TMZ by GSCs. The uptake efficiency of PAMAM-PEG-Tf/TMZ was indicated by the frequency of Alexa Fluor 555-positive cells as examined under fluorescence microscopy. Fluorescent intensities were analyzed by Imagine J software. The GSCs were seeded into 96-well plates and incubated with 50 μM TMZ, PAMAM/TMZ, PAMAM-PEG-Tf/TMZ or PAMAM-PEG-Tf for different period, or different concentration PAMAM-PEG-Tf/TMZ for 48 h. Cellular proliferation was determined by 3-(4,5-dimethylthiazol-2-yl)-2,5-diphenyl tetrazolium bromide (MTT) assay (Sigma) and expressed as a percentage as compared with untreated cells.

### Heterotransplantation of GSCs into the mouse brain

The male BALB/c nude mice at 6–8 weeks of age were bred and housed in a specific pathogen free animal facility. All animal experimental protocols were approved by the Institutional Animal Care and Use Committee of Soochow University and complied with the code of ethics for animal experimentation. The 10^5^ GSC cells were injected into the frontal lobe of the mouse cerebrum by stereotactic implantation to establish intracranial transplantation. The following experiments were carried out until brain tumors were formed.

### BBB permeability, uptake and cytotoxicity of PAMAM-PEG-Tf/TMZ in murine brain xenografts

PAMAM-PEG-Tf/TMZ solution (5mg/kg) was injected into the tail vein of mice that were implanted with intracranial glioma in a volume of 100 μl. Next, animals were anesthetized and sacrificed. Mouse brains were removed, and frozen in OCT embedding medium to prepare sections at a thickness of 10 μm. Sections were immunostained with anti-CD31 antibody to analyze BBB permeability of PAMAM-PEG-Tf/TMZ after 2 h and 12 h post-injection. The slides were stained by immunofluorescence to detect SOX2 protein expression in xenografts to assess uptake by GSCs, and TUNEL staining was carried out for analysis of cell apoptosis after 24 h post-injection.

### Evaluation of therapeutic potential *in vivo*

Mice were randomly divided into six experimental groups, and there were 10 mice in each group: Group 1, control; group 2, PAMAM-PEG-Tf; Group 3, TMZ; group 4, PAMAM/TMZ; group 5, PAMAM-PEG-Tf/TMZ; and group 6, PAMAM-PEG-Tf/TMZ . TfR+ cells were implanted with intracranial xenografts in groups 1 to 5, and TfR- cells were used in group 6. Drugs that included PAMAM/TMZ and PAMAM-PEG-Tf/TMZ containing 5mg/kg TMZ were injected into the tail vein of mice following brain tumor formation. TMZ was administrated by intra-gastric injection using a suspension at a dose of 20 mg/kg. Mice received drugs for 5 days continuously and were monitored daily until severe neurological deficits appeared. Survival analysis was used to compare the differences of each group according to survival time.

### Statistical analysis

Statistical analyses were carried out using SPSS version 19.0 (SPSS Inc., Chicago, IL, USA), and data was statistically determined by one-way ANOVA. The significance level was considered at an alpha value of P < 0.05. Each *in vitro* experiment was repeated at least three times. Overall mouse survivals that were implanted with intracranial glioma were estimated via Kaplan-Meier survival curves, and compared between groups via stratified log-rank tests. A Cox proportional hazards regression model was used to examine the validity of stratifying by groups and by factors.
